# Supplementary material for: Causal Relationship between Aging and Anorexia Nervosa: A White-Matter-Microstructure-Mediated Mendelian Randomization Analysis
Source: Biomedicines. 2024 Aug 16;12(8):1874. doi: 10.3390/biomedicines12081874 (PMC11351342; doi:10.3390/biomedicines12081874)
Supplement: Supplementary file 1 [file biomedicines-12-01874-s001.zip › Supplementary Figure/Figure S8/Leaveoneout plot of MR analysis for TL to ubm-b-1818.pdf]

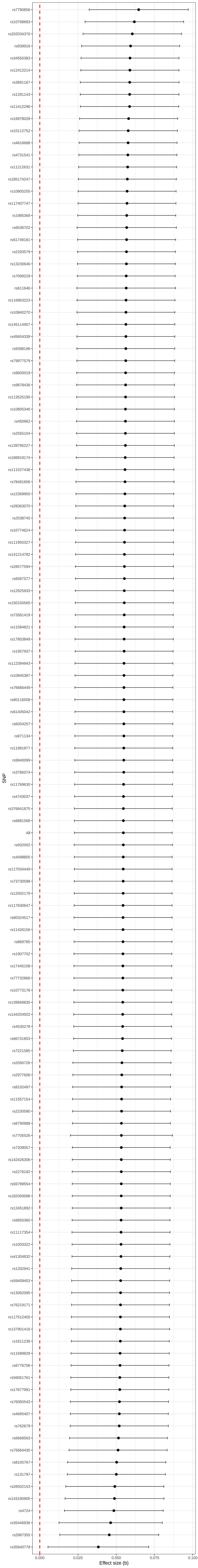
rs7790856
rs10768683
rs202034370
rs939916
rs34550383
rs12412214
rs3891167
rs1291143
rs11412296
rs16978028
rs10112752
rs4616688
rs4731541
rs11212631
rs185174247
rs10905255
rs117407747
rs1985369
rs6536702
rs61748181
rs2293579
rs13230646
rs7099229
rs611646
rs116863223
rs10840270
rs145114957
rs45604339
rs9398196
rs79977579
rs9600019
rs9878436
rs113525195
rs10805346
rs450962
rs2555104
rs139795227
rs188918174
rs111527438
rs78491606
rs12369950
rs28363070
rs2538745
rs10774624
rs111950327
rs141214782
rs28577594
rs6587577
rs12925933
rs150150565
rs73581419
rs11584821
rs17803849
rs1957937
rs112394943
rs10845387
rs76666449
rs80116508
rs61405042
rs6054257
rs871134
rs11991877
rs9940099
rs3785074
rs11769630
rs4743037
rs376641875
rs6881568
All
rs932002
rs4498805
rs117034449
rs73730598
rs12932179
rs117630647
rs80324517
rs11426156
rs869785
rs1907702
rs17445108
rs77732866
rs10773176
rs139669835
rs144204502
rs4530278
rs66731853
rs7221585
rs2056726
rs2977608
rs8102497
rs11557154
rs2230590
rs6790988
rs7705526
rs7209057
rs142426306
rs2276182
rs56799554
rs182059586
rs12451892
rs9955360
rs11117354
rs1003322
rs41304832
rs1332941
rs59409453
rs13062095
rs76219171
rs117512405
rs137901416
rs1611236
rs11699829
rs6776756
rs56061761
rs17677991
rs76065543
rs4695407
rs762679
rs6669563
rs75664430
rs8105767
rs131797
rs28502153
rs143190905
rs4724
rs35446936
rs2967355
rs35640778
SNP
0.000
0.025
0.050
0.075
0.100
Effect size (b)
